# The Extrachromosomal EAST Protein of *Drosophila* Can Associate with Polytene Chromosomes and Regulate Gene Expression

**DOI:** 10.1371/journal.pone.0000412

**Published:** 2007-05-02

**Authors:** Martin Wasser, William Chia

**Affiliations:** 1 Bioinformatics Institute, Department of Imaging Informatics, Singapore, Singapore; 2 Temasek LifeSciences Laboratory, National University of Singapore, Singapore, Singapore; 3 Department of Biological Sciences, National University of Singapore, Singapore, Singapore; Duke University, United States of America

## Abstract

The EAST protein of *Drosophila* is a component of an expandable extrachromosomal domain of the nucleus. To better understand its function, we studied the dynamics and localization of GFP-tagged EAST. In live larval salivary glands, EAST-GFP is highly mobile and localizes to the extrachromosomal nucleoplasm. When these cells are permeabilized, EAST-GFP rapidly associated with polytene chromosomes. The affinity to chromatin increases and mobility decreases with decreasing salt concentration. Deleting the C-terminal residues 1535 to 2301 of EAST strongly reduces the affinity to polytene chromosomes. The bulk of EAST-GFP co-localizes with heterochromatin and is absent from transcriptionally active chromosomal regions. The predominantly chromosomal localization of EAST-GFP can be detected in non-detergent treated salivary glands of pupae as they undergo apoptosis, however not in earlier stages of development. Consistent with this chromosomal pattern of localization, genetic evidence indicates a role for EAST in the repression of gene expression, since a lethal *east* mutation is allelic to the viable mutation *suppressor of white-spotted*. We propose that EAST acts as an ion sensor that modulates gene expression in response to changing intracellular ion concentrations.

## Introduction

The nuclear matrix as an internal non-chromosomal scaffold of the cell nucleus remains a controversial concept in eukaryotic cell biology [1]. It is poorly defined in terms of composition, function and subcellular localization. The EAST protein of Drosophila is a candidate component of an internal nuclear skeleton as it localizes to non-chromosomal regions and its overexpression can influence the size of this sub-nuclear domain [2]. A recent study demonstrated that EAST is found in the same protein complex as Megator (Mtor), a component of the nuclear pore complex and an ortholog of the mammalian TPR [3]. EAST and Mtor colocalize to another controversial structure called the spindle matrix [4]. Assembled from mainly nuclear proteins at prophase, the spindle matrix resembles a microtubule spindle. However, unlike microtubules, this structure is not destabilized by treatments that depolymerize microtubules. Hence the spindle matrix has been proposed to direct the formation of the bipolar mitotic spindle apparatus. Other potential components of the Drosophila spindle matrix include the nuclear proteins Chromator (Chro) and Skeletor that bind to the interbands of polytene chromosomes [5], [6]. Chro, also called Chriz [7], can interact with both Skeletor and Mtor. Interfering with the function of *Chro, Skeletor* and *east* has been shown to lead to defects in mitosis and meiosis [6], [8], [9]. The abundance of these four mentioned spindle matrix proteins in nuclei of postmitotic cells strongly suggests a vital function in interphase nuclei. Consistent with this idea, mutations in *Chro* affect the structure and organization of larval polytene chromosomes [10]. Mtor, Chro and Z4, an interactor of Chro, associate with a dosage compensation complex, indicating a role in transcriptional regulation [11].

In this report, we describe that the predominantly extrachromosomal EAST protein has the ability to associate with chromosomes and that this behavior is linked to a negative role in gene regulation.

## Results

### EAST can associate with chromosomes

**Figure 1:**
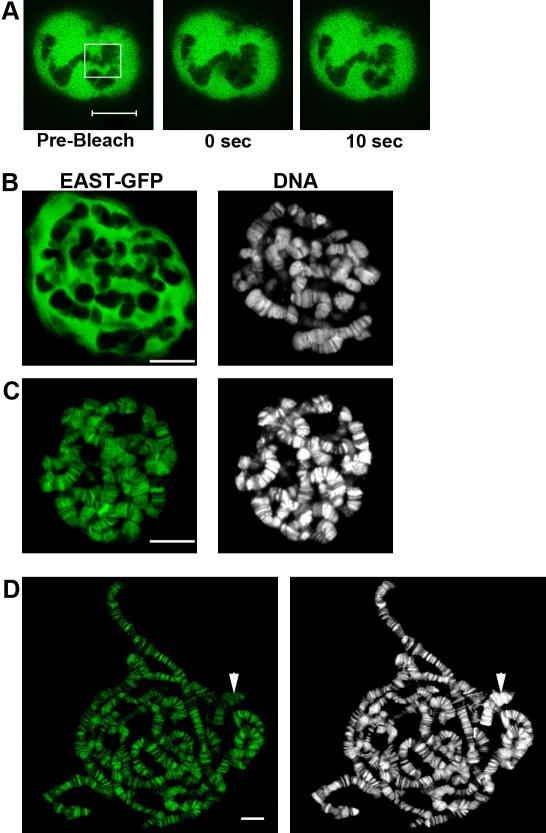
EAST can associate with Polytene Chromosomes. (A) Live intact salivary glands expressing EAST-GFP (green) were exposed to photobleaching. A rapid recovery (see Figure 3E) indicates a high mobility in the nucleoplasm. (B-D) Larval salivary glands expressing EAST-GFP (green) were fixed and counterstained with TOPRO3 (white) to visualize DNA. (B) In untreated cells, EAST-GFP is localized to extrachromosomal nuclear regions. (C) In larval salivary permeabilized with a saponin containing detergent buffer, EAST-GFP rapidly relocates to the polytene chromosomes. (D) In detergent treated and squashed preparations of polytene chromosomes, EAST-GFP shows a high degree of co-localization with DNA bands. The chromocenter is indicated (arrow head). EAST-GFP represents full length EAST with a C-terminal eGFP tag that was expressed by the GAL4 system using the *smid-Gal4* driver. Bars correspond to 10 µm in all panels.

We previously described EAST as a nuclear protein that localizes to an extra-chromosomal and extra-nucleolar compartment of the nucleus that we termed extrachromosomal nuclear domain (END) [2]. The expansion of the END in response to overexpression of EAST indicated that EAST might be modulating the extent of an interior nucleoskeleton. We expressed GFP-tagged EAST in 3^rd^ instar larval salivary glands and performed fluorescence recovery after photo bleaching (FRAP). An extremely fast recovery demonstrated that EAST-GFP is highly mobile, suggesting that EAST does not associate with a static internal nucleoskeleton (Figure 1A). For simplicity, we refer to the fusion protein containing the full-length version of EAST (residues 1-2301) with a C-terminal GFP tag as EAST-GFP.

To evaluate if EAST might associate with an ‘elusive’ structure termed the nuclear matrix, salivary glands expressing EAST-GFP were subjected to the first step of the Nuclear Matrix extraction protocol whereby cells are treated with detergent buffer. The permeabilization using saponin or Triton-X-100 results in a rapid re-localization of EAST-GFP onto polytene chromosomes (Figure 1C). An incubation of one minute is sufficient to deplete EAST-GFP from the END (Figure 1B) and relocate it to chromosomes (Figure 1C). The staining pattern is almost identical to that of chromosomal bands labeled by DNA dyes such as TOPRO-3, except in regions of the centromeric heterochromatin or chromocenter where the fluorescence intensity is significantly weaker relative to that of the DNA dye (Figure 1D). To rule out that the DNA binding was due to the GFP-part of the fusion protein, the protein trap line G180 [12] that is localized to the nucleoplasm was permeabilized. The G180 fusion protein was removed from the nucleus, demonstrating that the binding of chromosomes is linked to the EAST portion of the fusion protein (not shown).

**Figure 2:**
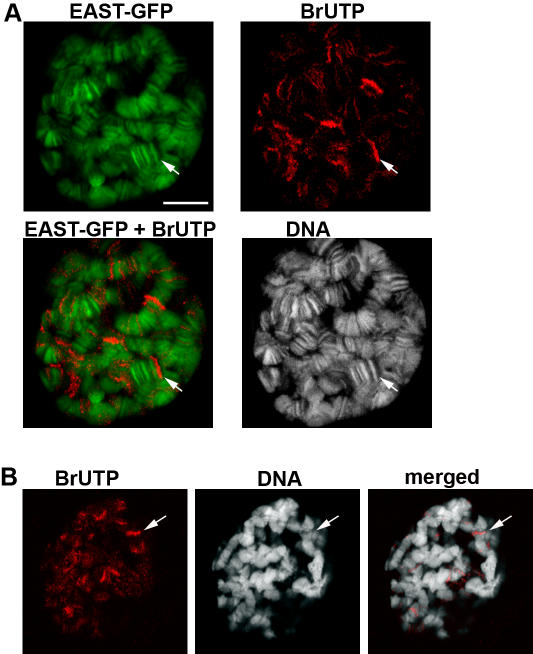
EAST-GFP accumulates in chromosome regions of low transcriptional activity. (A) EAST-GFP (green) is excluded from highly transcribed chromosome regions (arrow) in detergent treated larval salivary glands cells. Run-on transcription was visualized using BrUTP (red). TOPRO3 (white) was used to label DNA. (B) In *east* ^hop7^ mutants, like in wildtype, regions of high BrUTP incorporation (arrow) are found in interbands. Note that the banding pattern shows less contrast compared to EAST-GFP overexpressing or wildtype (not shown) cells. Bars correspond to 10 µm in all panels.

To investigate the relationship between transcription and the binding of EAST to chromosomes, we performed a run-on transcription assay by including BrUTP into detergent buffer. Transcriptionally active regions that incorporate high levels of BrUTP contain low levels of EAST-GFP (Figure 2A), while DNA bands showing strong EAST-GFP signals do not coincide with chromosome regions of high transcriptional activity. This localization pattern suggests that EAST preferentially binds to condensed and silent chromatin. To test if the removal of EAST could lead to a higher transcriptional activity in chromosome bands we performed a run-on transcription assay in *east* ^hop7^ mutants (Figure 2B). Loss of *east* results in the decrease of contrast in the banding pattern in polytene chromosomes. Despite the apparent disruption of chromatin structure, high incorporation of BrUTP is restricted to a few discrete, weakly condensed DNA regions, indicating that loss of *east* function does not result in a general derepression of transcription in chromosome bands. Furthermore, a subset of cells in permeabilized tissues incorporated fluorescently labeled BrdUTP, indicating that the experimental conditions did not block replication (not shown).

**Figure 3:**
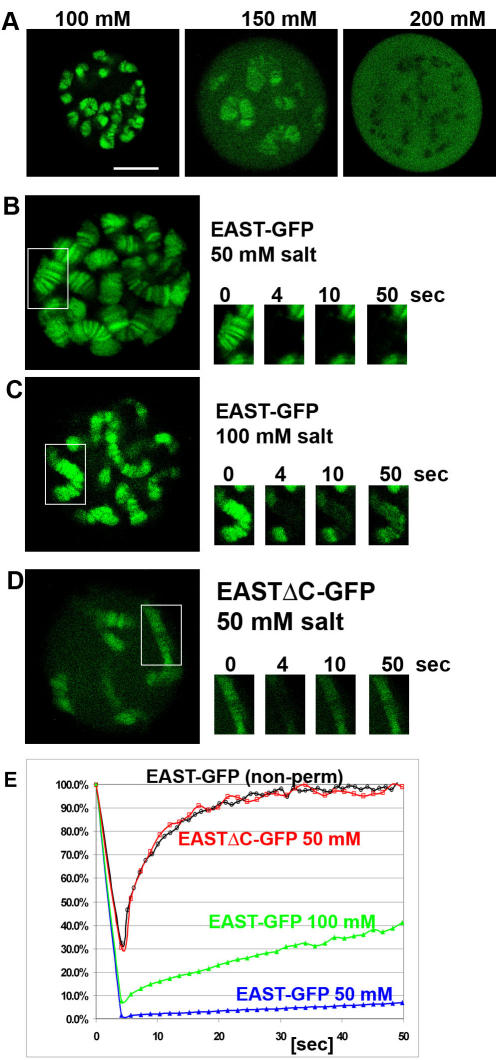
Characterization of EAST-GFP localization and mobility in larval salivary glands. (A) Varying the salt concentration can modulate localization of EAST-GFP. At 100 mM, the distribution is mostly chromosomal, at 150 mM chromosomal and nucleoplasmic and at 200 mM no chromosomal-like pattern is detectable. (B-D) The mobility of EAST-GFP was assessed by FRAP. (B, C) Increasing the salt concentration lowers the affinity to chromatin. An increase in salt from 50 mM (B) to 100 mM (C) leads to a faster recovery of fluorescence after bleaching chromosome regions bound by EAST-GFP. (D) Removing the C-terminal residues 1535-2301 of EAST leads to an increase in mobility. At a salt concentration of 50 mM, the truncated version of EAST associates with a lower affinity than the full-length version. (E) The diagram shows the recovery in seconds after bleaching the indicated nuclear regions for 4 seconds at a laser intensity of 100%. The two different variants of EAST-GFP were expressed using the *ftz-GAL4* driver. Cells were permeabilized in buffers containing 50 mM NaCl supplemented with varying amounts of KCl to reach the indicated salt concentrations. The recovery of the non detergent treated cell (non-perm) in Figure 1A is indicated for comparison. The Bar in A represents 10 µm and applies to all panels.

The affinity of EAST-GFP to chromatin depends on the salt concentration (Na^+^ or K^+^ ions) in the detergent buffer. At 100 mM or lower concentrations, the bulk of EAST-GFP associates with polytene chromosomes (Figure 3A). Increasing the concentration of either NaCl or KCl leads to a gradual dissociation from chromatin. Above 150 mM, which is the physiological salt concentration [13] fusion protein is increasingly found in the nucleoplasm. The dissociation is reversible as cells first incubated in 200 mM and then shifted to 100 mM salt will show chromosomal localization. In FRAP experiments, we observed a 10% recovery in 50 mM compared to a 40% recovery in 100 mM salt in the first 50 seconds after bleaching (Figure 3B, 3C and 3E), suggesting that increasing the salt concentration leads to a higher mobility of EAST-GFP bound to chromosomes.

FRAP was also used to compare the chromatin affinity of full length (residues 1-2301) and C-terminally deleted EASTΔC-GFP (residues 1-1520) at 50 mM salt. The truncated version of EAST was able to associate with polytene chromosomes, yet the banding patterns was less pronounced (Figure 3D). Moreover, fluorescence was also seen in the END. The very rapid and almost complete recovery of fluorescence in less than a minute indicates that the deletion of C-terminal residues 1520-2301 severely diminishes the affinity to chromatin (Figure 3E).

### Genetic evidence linking *east* to gene expression

**Figure 4:**
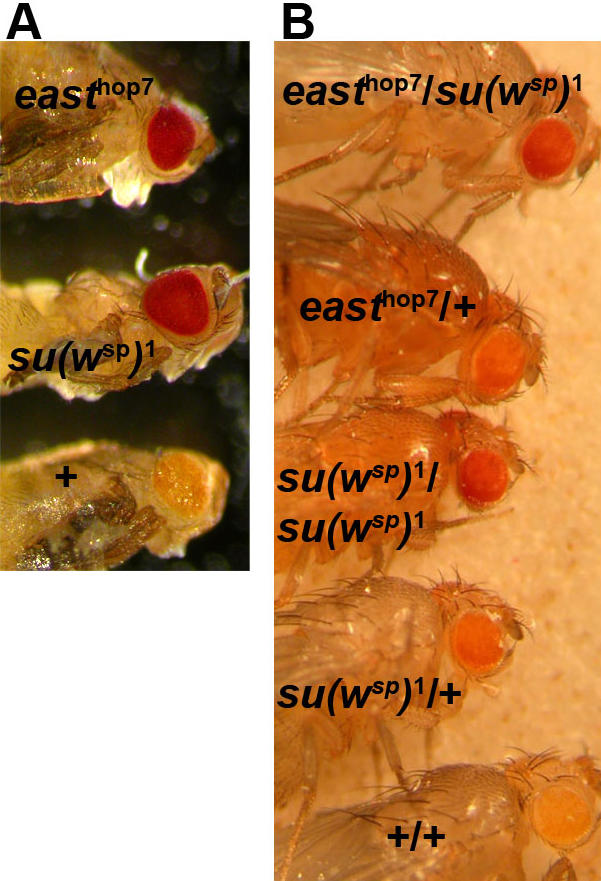
east corresponds to *suppressor of white-spotted.* All flies shown are homozygous for *w* ^sp1^. (A) In male pharates dissected out of pupal cases, the mutations *east* ^hop7^ and *su(w* ^sp^ *)* ^1^ suppress the loss of eye pigmentation resulting from the white mutation *w* ^sp1^. (B) The mutations *east* ^hop7^ and *su(w* ^sp^ *)* ^1^ show complementation. Like *su(w* ^sp^ *)* ^1^/*su(w* ^sp^ *)* ^1^, the combination *east* ^hop7^/*su(w* ^sp^ *)* ^1^ restores eye pigmentation to almost wildtype levels. Heterozygous flies of each mutation display intermediate eye pigmentation.

We learned that the sequence of *east* was identical to that of a gene called *suppressor of white-spotted* or *su(w* ^sp^ *)* (M. Belanich, personal communication). The *su(w* ^sp^ *)* mutation suppresses the reduction of eye pigmentation caused by 4 spotted alleles of the *white* gene (*w* ^sp1-4^), which are characterized by lesions in the upstream region of *w* [14]. We confirmed that *east* suppresses the *w* ^sp ^phenotype. Like the viable mutation *su(w* ^sp^ *)* ^1^, the pupal lethal allele *east* ^hop7^ restores eye pigmentation to almost wildtype levels (Figure 4A). The trans-heterozygous combination of *east* ^hop7^ and *su(w* ^sp^ *)* ^1^ is indistinguishable from homozygous *su(w* ^sp^ *)* ^1^ in terms of eye pigmentation (Figure 4B). Heterozygous *su(w^sp^)* ^1^ and *east* ^hop7^ females show intermediate levels of eye pigmentation in a *w* ^sp1^ homozygous background. Therefore, *east* and *su(w* ^sp^ *)* are allelic. To understand the molecular nature of the *su(w* ^sp^ *)* ^1^ mutation, we sequenced the coding region of the genomic DNA of the *east* locus. At codon 1956, we found a point mutation that introduces a premature stop codon, reducing the length of EAST protein from 2301 to 1955 amino acids. In summary, as has already been inferred from the genetic interactions between *w^sp^* and *su(w* ^sp^ *)*, the product of the *east/su(w* ^sp^ *)* gene might act as a negative regulator of gene expression that binds to the upstream regulatory region of the *w* gene.

### Chromosome association of EAST-GFP in apoptotic Cells

**Figure 5:**
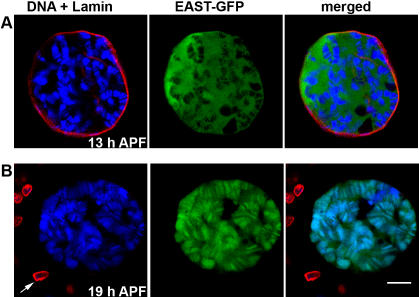
Change of localization of EAST-GFP during apoptosis. (A) In a 13 h APF old pupa, EAST-GFP (green) shows mainly extrachromosomal localization. The nuclear lamina (red) is still intact. DNA (blue) was labeled with TOPRO-3. (B) Co-localization of EAST-GFP with intact polytene chromosomes can be observed in a 19 hour APF old pupa. The destruction of the lamina indicates that part of the apoptotic program is executed. Note the barely detectable anti-lamin staining in salivary glands compared to neighboring diploid cells (arrow). The Bar in A represents 10 µm and also applies to B.

The intra-nuclear localization of EAST-GFP depends on the developmental stage. The fusion protein shows a mainly extrachromosomal pattern throughout larval and prepupal development (Figure 5A). During the early pupal stage at 18-19 hours after puparium formation (APF), we were able to detect cells showing a predominantly chromosomal localization (Figure 5B). This stage coincides with the time when salivary glands normally undergo apoptosis, which has been reported to be complete at around 16 hours APF [15], [16]. In a parallel study, we found that overexpression of EAST-GFP attenuates some but not all aspects of apoptosis (Wasser et al., unpublished). Salivary glands of *smid-GAL4 UAS-eastFL-GFP* pupae contained two types of cells: small cells lacking EAST-GFP with fragmented and condensed chromosomes and larger cells maintaining high levels of EAST-GFP expression, in which polytene chromosomes appeared to have escaped from apoptotic fragmentation. As has been previously reported, cell death in salivary glands is preceded by changes in the levels and localization of filamentous actin and nuclear lamin [17]. Consistent with the initiation of apoptosis in EAST-GFP expressing cells that contain intact chromosomes, the nuclear lamina exhibited degradation and filamentous actin disappeared from the cell cortex. In those cells that showed an attenuation of apoptosis, EAST-GFP was localized in a predominantly chromosomal pattern (Figure 5B). In summary, chromosomal localization of EAST-GFP can be observed in permeabilized larval as well as in untreated pupal salivary gland cells, suggesting that permeabilization might mimic a physiological change that occurs during programmed cell death.

## Discussion

In this study we show that the nuclear EAST protein has the intrinsic ability to associate with chromosomes and that this behavior is linked to an involvement in gene regulation. A previous study postulated that the gene product of *su(w* ^sp^ *)/east* would inhibit *white* by interacting with its upstream regulatory sequences [14]. Consistent with this prediction we discovered that the EAST protein possesses the ability to bind polytene chromosomes. The accumulation of EAST-GFP in transcriptionally silent heterochromatin regions and the absence from chromosome regions with a high level of transcriptional activity further support a repressive role in gene expression.

Because the characterization of EAST protein was carried out in permeabilized cells under non-physiological conditions it might be argued that the association with polytene chromosomes is an experimental artifact. However, we were able to observe co-localization with DNA in untreated cells while they undergo apoptosis. Moreover, despite permeabilization, cells were still able to perform transcription and replication, showing that chromatin binding of EAST did not occur under conditions that caused a general block of nuclear functions.

The affinity to polytene chromosomes is sensitive to the salt concentration of the detergent buffer. We observed co-localization of the bulk of EAST-GFP with polytene chromosomes in permeabilized cells at concentrations at and below 100 mM salt, which is lower than the estimated physiological concentration of 150 mM. The release of EAST-GFP from chromosomes with increased salt concentrations (150 mM and above) is consistent with the observation that the fusion protein does not display a discernible chromosomal localization pattern in intact cells.

The C-terminal residues 1520 to 2301 promote affinity to chromatin but are dispensable for nuclear import. This domain is also involved in tethering EAST to a nuclear remnant during mitosis [9]. A smaller segment of the C-terminus stretching from residues 1956 to 2301 is deleted in the *su(w* ^sp^ *)* ^1^ allele, suggesting that this region is required to mediate the suppression of pigmentation seen in the *w* ^sp^ alleles. The C-terminus therefore might promote binding to chromatin or recruit other factors involved in repression. Although the N-terminal residues 1-1955 seem to be deficient in gene silencing, they are sufficient for viability.

On the molecular level, the exact role of EAST in gene regulation remains a mystery. Two previous studies showed that the expression levels of *white* transcripts do not differ between wildtype and the allele *w* ^sp^ despite the lesions in the promoter regions [18], [19]. In microarray experiments, we did not detect significant differences in *white* transcription between *su(w* ^sp^ *)* ^1^ *w* ^sp1^ and *w* ^sp1^ adults, confirming the conclusion of the two earlier studies that altered *white* transcription does not appear to be responsible for the reduction in eye pigmentation (Wasser and Chia, unpublished). To test if sequence variations in *white* transcripts might explain the difference in pigmentation we sequenced cDNAs corresponding to *white* transcripts derived from *w* ^sp1^ and *su(w* ^sp^ *) w* ^sp1^ adults. However, we were unable to detect any evidence for genomic mutations or RNA editing.

**Figure 6:**
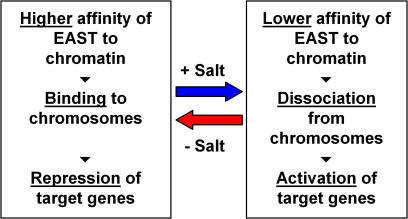
EAST might act as a salt sensor that modulates gene expression in response to changing ion (Na^+^/K^+^) concentrations. A change in EAST dosage may lead to the sensing of incorrect ion levels, which may result in inappropriate physiological responses.

Taken together, our results suggest that EAST could act as an ion sensor that fine-tunes the expression of target genes in response to fluctuating intra-cellular salt concentrations (Figure 6). Assuming a free nucleoplasmic and a bound chromosomal pool of EAST, the decrease in salt should shift the equilibrium towards the bound pool, resulting in down-regulation of target genes. Conversely, an increase in salt should relieve that repression. Target genes could be involved in maintaining cellular homeostasis, for instance by restoring physiological ion concentrations. This model would also predict that changing the protein concentration might mislead cells into perceiving an incorrect ion concentration. The inaccurate perception of cellular state could explain the increased sensory threshold to olfactory and gustatory stimuli that was observed in hypermorphic *east* mutants [20].

In a previous study we proposed that the increase in nuclear volume in response to EAST overexpression was caused by an expansion of a non-chromosomal nucleoskeleton [2]. An alternative interpretation might be that an ion influx could lead to hypertonic conditions of the cellular interior relative to the extra-cellular environment, resulting in nuclear swelling. On the contrary, a decrease in the intracellular potassium concentration is an early event in apoptosis and is required for optimal enzymatic activation of caspases and nucleases [21]. This decrease might explain the binding of EAST-GFP to chromosomes observed in cells undergoing apoptosis. During metamorphosis, the loss of *east* function leads to an acceleration of cell death in muscles, while overexpression of EAST-GFP in salivary glands attenuates apoptosis (Wasser et al., unpublished). These two opposite phenotypes could be explained by EAST counteracting the loss of K^+^ ions during apoptosis.

## Materials and Methods

### Drosophila Stocks

The *east* alleles used have been previously described [2], [20]. Canton-S (CS) was used as a wildtype strain. The UAS-GAL4 system [22] was used for salivary gland expression of transgenes. The effector construct lines *UAS-eastFL-GFP* and *UAS-eastΔC-GFP* have been described [9]. The salivary gland specific GAL4 driver lines *ftz-GAL4* and *smid-GAL4* [23] were provided by C. Doe and D. Shepherd, respectively. *w* ^sp1^ and *y* ^2^ *sc* ^1^ *su(w* ^sp^ *)* ^1^ *w* ^sp1^ were obtained from the Bloomington *Drosophila* stock center.

### Permeabilization and Imaging of Larval Salivary Glands

Permeabilization of larval salivary glands was performed using detergent buffer (0.3 M sucrose, 10 mM HEPES pH 7.3, 50 mM NaCl, 3 mM MgCl_2_, 1 mM EGTA, 2 mM DTT, 50 µg/ml saponin, 20 µ/ml of 1 Roche EDTA free protease inhibitor cocktail dissolved in 1 ml water) that was based on the cytoskeleton extraction buffer [24]. The salt concentration was increased by adding KCl to working concentrations of up to150 mM. For subsequent immunofluorescence staining, permeabilized tissues were fixed for 15 minutes in detergent buffer containing 4% formaldehyde (EM grade) without DTT and protease inhibitors (DB-fixative). To prepare squashes of polytene chromosomes, larval salivary glands were incubated for 2 minutes in detergent buffer, fixed for 3 minutes in DB-fixative and 2 minutes in 50% acetic acid, 4% formaldehyde. Spreading and squashing of chromosomes was performed as previously described [25].

The labeling of nascent RNA was adapted from a published protocol [26]. In brief, larval salivary glands were dissected in Ringer's solution, incubated for 15 minutes in detergent buffer supplemented with 0.2 U/µl RNase inhibitor (RNasin, Promega), 2 mM ATP, 1 mM CTP, 1 mM GTP (Roche) and 1 mM BrUTP (Sigma) and fixed in DB-fixative. BrUTP incorporated into nascent RNA was detected by indirect immunofluorescence using a monoclonal anti-BrUTP antibody (MAB3424, Chemicon). The labeling of run-on transcription sites could be inhibited by adding α-amanitin (Sigma) at a concentration of 100 µg/ml.

The mobility of GFP tagged proteins was assessed by fluorescence recovery after photo bleaching (FRAP) [27]. Permeabilized glands were transferred to a drop of detergent buffer between two 18×18 mm coverslips on a glass slide. Imaging and FRAP was performed by confocal microscopy after covering the tissues with a 22×32 mm coverslip. GFP was bleached for 4 seconds using an argon 488 nm laser light at an intensity of 100%.

### Immunocytochemistry

Antibody staining of whole-mount 3^rd^ instar larval salivary glands was carried out as previously described [2]. DNA was labeled using TOPRO3 (1∶5000, Molecular probes), nuclear lamina using anti *Drosophila* lamin Dm0 (1∶50, Developmental Studies Hybridoma bank, Paul Fisher). Images of fluorescently labeled samples were acquired by laser scanning confocal miscroscopy using the Zeiss LSM 510 Meta or Zeiss LSM 510 upright microscopes. Image processing was carried out using Adobe Photoshop, Zeiss LSM 5 image browser and Metamorph (Molecular Devices).
